# HealthyRHearts - reducing cholesterol in rural adults via telehealth-based medical nutrition therapy: protocol for a cluster randomised controlled trial

**DOI:** 10.1186/s12872-023-03306-8

**Published:** 2023-06-12

**Authors:** Tracy L. Schumacher, Jaimee Herbert, Jennifer May, Shanthi Ramanathan, Leanne J. Brown, Michelle Guppy, Annabelle Williams, Megan E. Rollo, John Attia, Clare E. Collins

**Affiliations:** 1 Department of Rural Health, University of Newcastle, Newcastle, Australia; 2 Food and Nutrition Research Program, Hunter Medical Research Institute, New Lambton Heights, Australia; 3 School of Health Sciences, College of Health, Medicine and Wellbeing, University of Newcastle, Newcastle, Australia; 4 School of Medicine and Public Health, College of Health, Medicine and Wellbeing, University of Newcastle, Newcastle, Australia; 5 Health Economics and Impact, Hunter Medical Research Institute, New Lambton Heights, Australia; 6 School of Rural Medicine, University of New England, Armidale, Australia; 7 Hunter New England Central Coast Primary Health Network, Broadmeadow, Australia; 8 Curtin School of Population Health, Faculty of Health Sciences, Curtin University, Bentley, Australia; 9 Heart and Stroke Research Program, Hunter Medical Research Institute, New Lambton Heights, Australia

**Keywords:** Cardiovascular disease, Medical nutrition therapy, Telehealth, Rural health

## Abstract

**Background:**

Few randomised controlled trials specifically focus on prevention in rural populations. Cardiovascular disease (CVD) contributes to approximately one quarter of deaths in Australia. Nutrition is a key component affecting many risk factors associated with CVD, including hypercholesterolaemia. However, access to medical nutrition therapy (MNT) is limited for people living in rural areas, potentially exacerbating inequities related to health outcomes. Telehealth services present an opportunity to improve MNT access and address healthcare disparities for rural populations. The present study aims to evaluate feasibility, acceptability, and cost-effectiveness of a telehealth MNT CVD intervention program in lowering CVD risk over 12-months in regional and rural primary health care settings.

**Methods/design:**

A cluster randomised controlled trial set in rural and regional general practices in NSW, Australia, and their consenting patients (n = 300 participants). Practices will be randomised to either control (usual care from their General Practitioner (GP) + low level individualised dietetic feedback) or intervention groups (usual care from their GP + low level individualised dietetic feedback + telehealth MNT intervention). Telehealth consultations will be delivered by an Accredited Practising Dietitian (APD), with each intervention participant scheduled to receive five consultations over a 6-month period. System-generated generic personalised nutrition feedback reports are provided based on completion of the Australian Eating Survey – Heart version (AES-Heart), a food frequency questionnaire. Eligible participants must be assessed by their GP as at moderate (≥ 10%) to high (> 15%) risk of a CVD event within the next five years using the CVD Check calculator and reside in a regional or rural area within the Hunter New England Central Coast Primary Health Network (HNECC PHN) to be eligible for inclusion. Outcome measures are assessed at baseline, 3, 6 and 12 months. The primary outcome is reduction in total serum cholesterol. Evaluation of the intervention feasibility, acceptability and cost-effective will incorporate quantitative, economic and qualitative methodologies.

**Discussion:**

Research outcomes will provide knowledge on effectiveness of MNT provision in reducing serum cholesterol, and feasibility, acceptability, and cost-effectiveness of delivering MNT via telehealth to address CVD risk in rural regions. Results will inform translation to health policy and practice for improving access to clinical care in rural Australia.

**Trial registration:**

This trial is registered at anzctr.org.au under the acronym HealthyRHearts (Healthy Rural Hearts), registration number ACTRN12621001495819.

## Background

A person’s choice to live in a rural or metropolitan area can affect their cardiovascular health [1–6]. The reasons for this are multifactorial and comprise a range of environmental and societal factors including availability and access to appropriate services and healthcare professionals [1–3, 7]. At a personal level, modifiable and non-modifiable risk factors for cardiovascular health in rural residents may include sex, age, Indigenous status, socioeconomic status, education, health literacy, physical activity, access to healthy foods and food insecurity [4–6].

A meta-analysis of international published data has shown that cardiovascular disease (CVD) related lipids are worse in urban populations compared to rural [8]. However, in Australia, it has been shown that if modifiable risk factors for CVD in rural populations were equal to those in metropolitan areas, the attributable death rate would be reduced by 38% [9]. These modifiable risk factors include diet, alcohol, smoking behaviours, physical activity and body mass index (BMI) [9]. While rural Australian populations have been reported to have higher vegetable intakes, other risk factors, such as prevalence of smoking, alcohol consumption, saturated fat and total energy intakes are significantly higher compared to urban counterparts [9].

Whilst there is a need to privilege preventative medicine addressing CVD risk factors [10], there is little published research specific to rural communities to guide practice [11]. Specifically, there is limited evidence of improvement in dietary intake patterns secondary to nutrition interventions in the context of CVD prevention and management within rural communities [12–14]. In addition, only a small proportion of research funding is dedicated to rural research [15]. Existing research has uncovered many challenges [4], differences and similarities in risk factors stratified by location [4, 8, 9], and some information on service delivery models for chronic health outcomes in rural populations [16].

Improving nutritional intake is a prevention strategy that can affect multiple risk factors for the development of CVD, thus The National Vascular Disease Prevention Alliance Absolute Risk Guidelines recommend dietary intervention for CVD prevention and treatment [17, 18]. There is also evidence that individuals are more likely to improve their dietary patterns if they receive personalised nutrition assessment and feedback, and regular, ongoing support from an Accredited Practising Dietitian (APD) [19]. However, dietitians have rarely been used in either the development or delivery of nutrition interventions [13], and translation techniques are not adequately described [20]. In addition, access to APDs in rural and regional areas who are trained to provide medical nutrition therapy (MNT) is challenging. Barriers include remote location of patients, long travel times, long waiting lists, consultation costs, reimbursable care plan restrictions, inadequate referral pathways, and fewer service providers.

Clinicians and patients need access to MNT tools that are fit for purpose, valid and engaging. This includes detailed assessment of usual dietary patterns, and identifying aspects related to CVD. Many, including the general population and people working in the healthcare system, are unaware that improved access to APD services can improve diet related health and patient outcomes [21]. The result is regional and rural patients’ risk factors remain unchanged or worsen, while reliance on medications, healthcare burden and mortality risk escalate [22].

## Methods

The current study is a parallel group 12-month cluster randomised controlled trial (RCT) that compares provision of two levels of dietary advice within a rural primary care setting. Primary care practices are randomised such that patients in the first group will receive usual care as provided by their General Practitioner (GP), with the addition of a dietary assessment via a food frequency questionnaire (FFQ) which includes an individualised automated feedback report on usual eating patterns. The second group will also receive individualised feedback from the FFQ, and an additional five telehealth MNT consultations over a six-month period with an APD.

The aim of this trial will be to reduce serum lipids associated with CVD health. The secondary aim will be to reduce modifiable risk factors related to CVD health, such as blood pressure, anthropometry and improve dietary patterns based on the proportion of total energy contributed by foods from food groups that are energy-dense, nutrient-poor and also those that are nutrient-dense.

### Study settings

The trial is set in primary care practices, in rural or remote areas covered by the Hunter New England Central Coast Primary Health Network (HNECC PHN) [23]. Rural location is defined here using the Modified Monash Model (MMM) [24]. MMM categorises an area as city (MM 1), regional (MM 2), rural (MM 3–5) or remote (MM 6–7). Areas included in this trial are categorised as MM3 or higher, ranging from towns with 15,000–50,000 people, to very remote locations.

### Ethical and research governance approval

This project was ethically approved by the University of Newcastle Human Research Ethics Committee (H-2021-0193), with additional safety approvals from the University of Newcastle Health and Safety Committee (49/2021). This trial was also registered with the Australian New Zealand Clinical Trials Registry on 3rd November 2021 (ACTRN12621001495819). Protocol amendments will be updated on this site as they occur and will be approved by the above stated ethics committee. Relevant people will be notified of amendments in accordance with the process approved by the ethics committee. Written informed consent was obtained from all study participants. This study was conducted in accordance with the Australian Code for the Responsible Conduct of Research.

### Study participants and eligibility

Two different types of participants are to be recruited to this study:

1. General practices / General Practitioners (GPs). General practices or GPs that are based within the HNECC PHN footprint and that are categorised within MM 3–6 are eligible to be recruited. Practices must also have a Pen CS Clinical Audit Tool (PENCAT) agreement with the Primary Health Network. PENCAT is a clinical audit tool, available to Australia’s primary care practices through the PHN [25]. The PENCAT Plus clinical suite can be used by Best Practice or Medical Director practice management software. The practice must operate at least one day per week in the rural area identified to be eligible for registration. At the time of project development (9th Feb 2021), 76.8% of practices in the target group were using PENCAT software. General Practices and GPs who consent to the study must agree to providing usual care to any of their study participants for the 12 months they are involved in the study.

2. Patients of the registered GPs. Eligible patients must live within an area rated as MM 3 or higher in the HNECC PHN footprint and be assessed by their GP as being at moderate to high risk (≥ 10%) of a CVD event over the next 5 years according to the Framingham Risk Equation, the CVD risk calculator, or using clinical judgement and referring to the Guidelines for the management of Absolute cardiovascular disease risk [17, 26].

A person may be eligible for inclusion if they have no known coronary artery disease (CAD) or they are judged by their GP to be currently stable with a CAD diagnosis and free of clinical events for at least 6 months. People are ineligible for the study if:

- Their GP considers them to be ineligible due to the complexity of their condition or is aware of a circumstance that would impact on their ability to participate in the study.
- They have a medical condition that affects dietary intake, e.g., conditions with swallowing difficulties, or restrictive exclusion diets; they are newly diagnosed with diabetes (< 3 months).
- They are unable to participate in telehealth dietary consultations due to disability or medical condition.
- They have been hospitalised or revascularized in the preceding 6 months.
- They have not been on a stable statin dose for the preceding 3 months.
- They do not have access to an email address or to the internet.

### Interventions

**Fig. 1:**
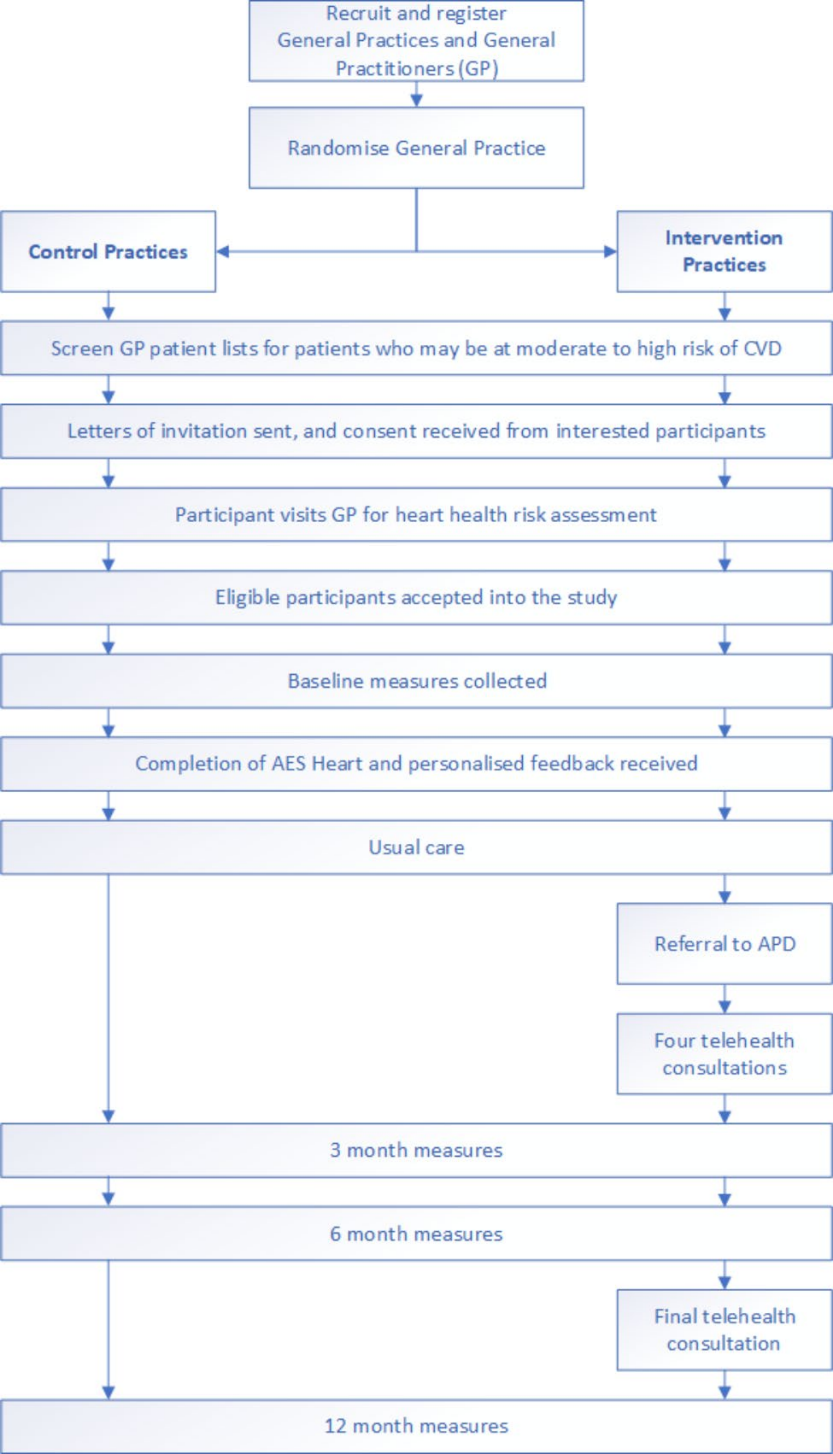
Design of the intervention

The overall design of the intervention can be seen in Fig. 1.

#### Usual care / control group

All participants will receive usual care as determined by their GP, regardless of randomisation status. This includes the management of any risk factors identified as part of the intervention or that may present over time. Additionally, all participants in the study are invited to complete the AES-Heart at baseline, 3-, 6- and 12-months. The AES Heart is an online 242 question Food Frequency Questionnaire (FFQ), that asks about usual consumption in the last 3–6 months. The AES-Heart is based on the Australian Eating Survey (AES), with additional questions added relating to foods significant to heart health. Both the AES and AES-Heart are validated for usual intake [27, 28]. Upon completion of the FFQ, the user is given immediate visual and written feedback that compares their nutritional intake to national dietary and heart health guidelines.

#### Intervention group

All participants in the intervention group will receive usual care from their GP, and the AES-Heart personalised nutrition report at the same times as the usual care group. In addition to usual care, they will also receive two hours of MNT dietetic consultations from an Accredited Practising Dietitian (APD), via a telehealth methodology over five separate appointments. The primary method of consultation will occur via *healthdirect* Video Call [29], an Australian telehealth tool that complies with the Australian Privacy Policy and legal requirements for managing medical data. When internet coverage is insufficient, a telephone call is substituted.

The baseline dietetic appointment of 30 min is scheduled as soon as possible, with the next three appointments following at + 2, + 4 and + 12-week intervals. The final dietetic consultation at 6 months is of 30-minute duration. During these sessions, APDs use a suite of existing behavioural nutrition tools previously developed and adapted for a regional and rural setting and for CVD prevention [30, 31]. A summary of the session including action plans and relevant resources is sent to the participant via email following each session. A brief summary of topics covered in consultation is detailed in Table 1.

**Table 1:** Brief summary of dietetic consult topics and timing

Consultation	Timing	Topics covered
1	30 min @ baseline	Discussion of AES-Heart and PNQ report, negotiation of SMART goals, provision of necessary resources
2	20 min @ baseline + 2 weeks	Review of SMART goal progress and discuss barriers and enablers to achieving goals, negotiate any necessary changes and provide necessary resources.
3	20 min @ baseline + 4 weeks	Review of SMART goal progress and discuss barriers and enablers to achieving goals, negotiate any necessary changes and provide necessary resources.
4	20 min @ baseline + 12 weeks	Review of SMART goal progress and discuss barriers and enablers to achieving goals, review 12-week AES-Heart report, negotiate any necessary changes and provide necessary resources.
5	30 min @ 6 month	Review of SMART goal progress and discussion of progress through intervention, review final AES-Heart report and conclude sessions.

The behaviour change theoretical framework underpinning the MNT dietetic intervention is Michie’s behaviour change paradigm [32]. This behaviour change wheel is a non-linear classification that incorporates previously identified behaviour change intervention characteristics into a system of Capability, Opportunity, Motivation – Behaviour (COM-B) attributes.

Prior to their first consultation, participants are asked to complete an online Personalised Nutrition Questionnaire (PNQ) [30, 33, 34] to identify COM factors that an individual participant believes to be impacting on their ability to usually eat healthily. During the consultation, the APD will be able to use the both participant’s PNQ and AES-Heart report, along with appropriate resources to direct and tailor discussion around goal setting, nutrition interventions, and behaviour change. A full description of the development of the intervention has been submitted for publication elsewhere.

#### Health professionals delivering the intervention

The MNT intervention will be delivered by APDs. Dietitians wishing to practice privately in Australia must hold APD status with Dietitians Australia [35], thus an APD qualification requirement ensures the study is consistent with the current healthcare setting standards. APDs employed in the study must have completed continuing professional development modules that cover telehealth practice, and CVD nutrition. They must also work in a rural area classified as MM 3–6 or be able to demonstrate they have provided continuity of care to a rural community within the previous five years. Employed APDs will also have the capacity to provide continuous care over the course of the participant’s five scheduled consultations. Participants will have the same APD throughout their five consultations, unless a change is requested by the participant, in order to assist in building rapport with participants and ensure that advice pertinent to the participants’ situation is provided.

### Study outcomes

The primary study outcome is change from baseline in total serum cholesterol, as collected and analysed by National Associate of Testing Authority accredited pathology services in the state. Data for this outcome is collected at baseline, 3-, 6- and 12-months.

Secondary outcomes for the study include change from baseline for:

i) Serum low density lipoprotein (LDL) cholesterol, collected at baseline, 3-, 6- and 12-months.
ii) Serum triglycerides, collected at baseline, 3-, 6- and 12-months.
iii) Systolic and diastolic blood pressure, collected at baseline and 12 months after randomisation.
iv) Percentage of total energy dietary intake derived from energy-dense, nutrient-poor and nutrient-dense food groups. This is measured by food frequency questionnaire and collected at baseline, 3-, 6- and 12-months after randomisation.
v) Self-reported weight. This data will be collected at baseline, 3-, 6- and 12-months.
vi) Self-reported waist circumference. This data will be collected at baseline, 3-, 6- and 12 months.
vii) Quality of life, assessed at baseline, 3-, 6- and 12-months.

### Power and sample size calculation

To detect a change in total cholesterol (primary outcome) of 0.51 mmol/L (SD = 1.1) [31], with 80% power and alpha 0.05, 74 people are needed. Allowing for a 25% drop out requires 100 people per group. To account for clustering by GP practice, we assume 10 practices per arm, each contributing 10 people. The design effect, assuming an ICC of 0.05 is 1.45, meaning a final sample size of 145–150 people per arm (15 practices per arm, each contributing 10 people) for a total of 300 patients is required.

### Randomisation

General Practices recruited to the project will be stratified to ensure samples are representative.

i) Practices will firstly be stratified by MM categories 3–5.
ii) Practices will also be stratified by practice size according to the following criteria: (1) small practice (1–5 GPs in the practice at the time of registration) and (2) medium to large practice (more than 5 GPs working in the practice at the time of registration).

A randomisation procedure for the practices was developed by a statistician external to the project, with practices being assigned to either the intervention or control arm. The randomisation module was uploaded into the research REDCap database, by an external REDCap administrator, so that all researchers are blinded until randomisation takes place. Practices and participating GPs will be unaware of the allocation of their group until at least one GP from the practice has registered. Other GPs from the same practice may register with the study after the allocation is known, for example when a new registrar joins the practice.

Recruitment of practices will continue until the target number of participants is met, or no further practices are available to be approached.

### Recruitment

#### GP registration

Initial approach will be from a study recruiting officer to the practice manager or person who acts in the practice manager role. Both the practice and individual GPs will be asked to register for the study. Following GP registration, the study recruiting officer will ask the practice to screen the GP’s current patient list in-house for potential study participants. Each one will then be vetted for suitability. General Practices will receive $100 payment for each participant who consents to participate and is subsequently randomised to the study. 

GPs may also opportunistically invite patients who present for appointments, by giving them a note to take to reception, where they are presented with a generic invitation to read later.

#### Participant recruitment

Potential participants will initially receive a letter of invitation from their GP and will be instructed to return a signed consent form to the researchers or make contact if they have any questions. Participant consent acknowledges that the GP will be sharing information about the person’s heart health with the study and that the study will be sharing pertinent data collected with the GP and provide options for consenting to ancillary analyses. All participants are assigned a unique study ID upon receipt of a consent form. After consent is received, the potential participant’s eligibility will be assessed and current medications for heart health noted. The participant will then be booked an appointment with their GP for the heart risk assessment. Once a heart health assessment has been completed, the GP will then forward the appropriate results to the study.

People who consent to participate but are ineligible to do so because their level of risk is categorised as LOW, may opt to complete the AES-Heart, as per all participants in the study and receive the individualised feedback.

### Data collection, management and analysis

#### Patient referrals to the study

The following minimum information is provided as part of the GP referral to the study to confirm the participant’s assessment as being at moderate or high risk for inclusion into the study: age, sex, smoking status, blood pressure (both current and unmedicated if applicable), lipids (both current and unmedicated if applicable) and diabetes status.

Family history of cardiovascular disease, kidney function, presence of familial hypercholesterolaemia, evidence of atrial fibrillation and any medications the person is currently taking that may be associated with their cardiovascular health or pertinent to a dietetic referral may also be provided to confirm the level of risk.

At the end of the study, any information for consenting participants on major adverse cardiac and cerebrovascular events will be sourced from the Cardiac and Stroke Outcomes Unit, the repository for all events of this type in the region.

### Data required post-inclusion into the study

#### Medications

Whilst prescribed medications will be sourced from GP referrals, participants will also be asked over the phone which medications they take, and their perceived adherence. This will be done at baseline, and participants will be asked about any change in medications via their online surveys at 3-, 6- and 12-months.

#### Biological samples

Fasting blood samples will be collected at baseline, 3-, 6- and 12-months to measure total cholesterol (main primary outcome), HDL and LDL cholesterol, triglycerides, electrolytes, urea and creatinine, liver function, glucose and HbA1c. Samples will be collected and analysed by an accredited pathology site.

#### Anthropometry and blood pressure

Height, weight, and waist circumference will be collected by the participant, using the instructions or videos given as reference guides. Each participant will be given a paper tape measure for measuring their waist circumference to the nearest 0.5 cm [36]. Waist will be measured at the top of the hip bone.

Weight is to be collected using own scales to the nearest 0.1 kg (or 100 g). Height will be measured using the paper tape measure to the nearest 0.5 cm. Where weighing scales are not available, participants will be asked to use the scales at their GP practice at their baseline and 12-month assessments.

Blood pressure will be measured by the GP, or their appropriate staff, at their service in accordance with their usual care and equipment. Values used are those taken as part of the heart health assessment, and unmedicated historic values if required.

#### Surveys and questionnaires

Surveys and questionnaires will be used to collect information on dietary intake, physical activity levels, quality of life, health literacy and other relevant demographics not captured in patient records.

### Dietary intake

Dietary intake will be measured by the AES Heart, a food frequency questionnaire that is an extended version of the adult Australian Eating Survey. The AES Heart captures additional details regarding fat and salt intakes, and foods or nutrients evidenced to reduce cholesterol. The AES Heart is an online tool that provides immediate personalised feedback on eating habits related to heart health. The AES Heart was previously validated for long-chain fatty acids and used to estimate dietary intake over the 3–6 months, reflecting time since last follow-up [28]. The version used in this study has been slightly modified to be administered online and provide system-generated immediate personalised nutrition reports and feedback.

### Physical activity

Physical activity (PA) will be measured using the Active Australia Survey [37]. The Active Australia Survey is used by the Australian Institute of Health and offers short and reliable questions to measure leisure time physical activity.

### Quality of life and sleep

Quality of life (QoL) will be measured using the PROMIS GLOBAL Health Scale v1.2 (13th April, 2018, Global 10), a series of 10 questions that aims to measure QoL experienced over the last 7 days [38]. Sleep will be measured using the PROMIS short form Sleep Disturbance 4a, a four-item questionnaire that scores sleep quality and compares it to a mean score of 50 [39].

### Health literacy and patient activation measures

Two health literacy tools will be used here. eHEALS is a validated measure of eHealth literacy for older adults [40]. It is an 8-item measure that questions the participants use of internet-based resources. The All Aspects of Health Literacy Scale (AAHLS) captures general health literacy [41].

The Patient Activation Measure (PAM) is a 10–13 item measure that aims to categorise participants according to their knowledge, skills and confidence in managing their own healthcare. It can be converted into a 100-point quantifiable scale that is predictive of patient engagement with their healthcare. Responses are categorised into four broad categories that range from “disengaged and overwhelmed” to “maintaining behaviours and pushing forward”. It is appropriate for use in older adults and can demonstrate how changes in category may also affect other aspects of life [42, 43].

To ensure congruency between clinicians and patients, registered GPs and APD’s will complete the Clinician Support Patient Activation Measure (CS-PAM) [44]. The CS-PAM mirrors the questions asked in PAM but is modified to be answered from the clinician perspective.

### Other demographic and medical data

Other demographic data that may impact on heart health will be collected from the participant via a survey, including social history, living arrangements, level of education, household income and the number of dependents.

At 12 months, participants will be contacted by telephone to book in for their annual heart health assessment. At this time, participants will be asked about any GP visits regarding their heart health care in the previous 12 months and any referrals to other health care professionals.

### Biological specimens

A sub-sample of consenting participants will be asked to provide additional biological samples for future genetic testing and to correlate responses to the AES-Heart. Participants are to be chosen based whether they are able to access a pathology collection and processing site that is able to process the samples in a time frame that protects the integrity of the samples and is able to freeze the samples at -80^o^C on site.

### Intervention feasibility

Semi-structured interviews will be conducted with APDs and consenting participants after the first 6-months of the intervention to test its feasibility. Interviews will be strengths-based and continue until theme saturation. Sampling for representative demographic or geographic factors, and a thematic analysis will be performed. For all groups, questions will focus on trial experience and include acceptability and appropriateness of approach for regional/rural patients, potential improvements, and opportunities for wider adoption of MNT for CVD.

### Intervention fidelity, barriers, and enablers

Audio recordings will be collected from a sub-sample of dietetic consultations throughout the intervention. These will be used to qualitatively assess APDs for intervention fidelity specifically the negotiation of participant goals, measures of success, and identification of barriers and enablers.

### Data management

REDCap will be used to collect and store research data [45, 46]. Data will be added directly by participants or by members of the management or recruiting team. Where possible, original records will be kept electronically, to allow for checks where data outliers are identified. Data is transferred securely to and from General Practices using any method approved by the ethics committee. The study Chief Investigator, Project Manager, Statisticians and Economic Analysts associated with the analysis may have access to the final dataset. The de-identified datasets used and / or analysed during the current study may be available from the corresponding author on reasonable request.

### Statistical methods

Linear mixed models will be used to analyse changes in outcomes, with a fixed effect for arm, and random effect for individual and cluster. The effect of the intervention will be based on the coefficient of the arm by time interaction term and adjusted for primary care service.

#### Economic analysis

A two-step economic analysis is planned. First, from the perspective of patients and health service providers, a cost-consequence analysis will compare both costs and consequences between usual care and the 6-month tailored MNT intervention. The analysis will be based on within-trial cost and outcome estimates. Costs will capture the resources required to deliver MNT from a provider and participant perspective. These resources will be compared against consequences that will include changes in quality of life, LDL cholesterol, dietary intake, and physical activity. The second analysis, from the perspective of patients and healthcare providers, will be a within-trial cost-effective analysis. The measure of effect will be health gain represented by quality-adjusted life years (QALYs) derived from Global 10. Results will be reported as an incremental cost-effectiveness ratio. A sensitivity analysis will be conducted to explore the robustness of the results to the uncertainty around the parameters. The economic results will be considered in the context of decision-making criteria that include strength of evidence; capacity of the intervention to reduce inequity; acceptability to stakeholders; feasibility; and sustainability.

#### Impact assessment

An impact evaluation of the overall study will be undertaken using the Framework to Assess the Impact of Translational health research (FAIT). FAIT is a comprehensive, multi-method impact assessment framework that assesses impact in quantitative, qualitative and economic terms. The modified Payback method will capture overall knowledge, capacity building, practice, policy, economic, social and health impacts of the research program. A program logic model featuring progress, outcome and impact metrics will be used to guide data collection and management procedures that underpin FAIT. A cost-effectiveness analysis will inform the economic assessment within FAIT that will aim to monetise the return on the research investment. Qualitative data collected during process evaluations will inform the ‘narratives of how translation occurred’ and describe impacts in the words of key stakeholders such as participants, APDs and GPs. All three impact assessment methods will inform a comprehensive assessment of the impact of this project.

## Methods: monitoring

### Data monitoring

Only the Project Manager, Dietetic Manager/PhD candidate, project assistant, recruiting officers and Chief Investigator have access to the full stored data set. APDs on the study have limited access to participant information. The Business Manager is limited to data relating to the General Practice administration fee. No separate data monitoring committee was established as the services provided are designed to mimic that of usual health care and participants remained under the care of their General Practitioner at all times. Dietetic interventions for prevention of heart disease are recommended as part of usual care [17]. However, this trial is required to report progress to the study funders at specified time points.

## Discussion

This cluster randomised controlled trial aims to use referral pathways in primary care to determine whether serum cholesterol can be improved following an addition of telehealth MNT to usual care in rural NSW in those at increased risk of heart disease. Limited evidence from high-quality nutrition interventions among rural communities addressing CVD risk exists [12]. Hence, the current trial will address this gap and add to the evidence base. It is expected that the series of personalised telehealth appointments with qualified APDs who have received additional training, will facilitate a decrease serum cholesterol among participants, and the therapeutic APD-participant interaction will aid long-term nutrition-related heart health behaviour change. Previous research in a small sample using a similar method of APD consultation found that diet quality could be improved, and total energy derived from energy-dense, nutrient-poor foods reduced by approximately 7% [30].

There are several practical and logistical concerns to be addressed. These include that participation in cardiovascular clinical trials has historically been low for women [47]. More importantly, the demand for GPs in Australia is currently increasing, and a shortfall has occurred, with reduced access particularly impacting people living in rural and remote areas [48]. A large research project conducted prior to the COVID19 pandemic with similar recruiting strategies indicated that the median number of participants randomised per GP was three [49]. This means that the current study will need to provide a high level of support to GPs to achieve the commitment necessary. Engaging practices and GPs and reaching the desired sample size in a time of high demand, e.g., in case of a severe flu season, is therefore likely to be challenging. One of the unintended consequences of implementing this project following a pandemic may be that lessons are learned regarding recruitment of rural GPs and their practice staff into research, during a period with complex time and resource pressures.

In addition, barriers to telehealth utilisation exist, particularly in rural areas and include both cultural and technological issues [50]. Previous research in the target region has shown that telehealth may be perceived as a lower level of care, or replacement of services by healthcare practitioners [51]. Additionally, those who had already used telehealth services reported technological barriers that diminished the quality of interactions [51]. However, recent evidence from other research studies providing allied health services to rural populations via telehealth indicated that overall experiences were positive [52].

## Conclusion

This rural telehealth project investigates the feasibility and efficacy of person-centred dietetic consultations, specifically designed for non-metropolitan areas of NSW. Insights from this project are likely to be applicable to other rural regions, and will emphasise that local context, knowledge and relationships are highly important when seeking to conduct nutrition-related telehealth research in rural areas of Australia.

## Data Availability

Not applicable.

## Abbreviations

AES-Heart: Australian Eating Survey – Heart version
APD: Accredited Practising Dietitian
CS-PAM: Clinician Support Patient Activation Measure
CVD: Cardiovascular disease
FAIT: Framework to Assess the Impact of Translational health research
GP: General practitioner
HNECC PHN: Hunter New England Central Coast Primary Health Network
LDL: Low-density lipoprotein
MNT: Medical Nutrition Therapy
MMM: Modified Monash Model
NSW: New South Wales
PAM: Patient Activation Measure
QALY: Quality-adjusted life years
